# MUSE-RASA captures human dimension in climate-energy-economic models via global geoAI-ML agent datasets

**DOI:** 10.1038/s41597-023-02529-w

**Published:** 2023-10-12

**Authors:** Diego Moya, Dennis Copara, Alexis Olivo, Christian Castro, Sara Giarola, Adam Hawkes

**Affiliations:** 1 Technology Outlook and Strategy, Technology Strategy and Planning Department, Saudi Aramco, Dhahran, 34465 Saudi Arabia; 2 Department of Chemical Engineering, Imperial College London, South Kensington, London, SW7 2BX UK; 3 Institute for Applied Sustainability Research, IIASUR, Quito, 170806 Ecuador; 4 Departamento de Comunicación e Información, Universidad Oberta de Catalunya, Avenida Tibidabo, Barcelona, 39-43 08035 Spain; 5 Carrera de Ingeniería Mecánica, Facultad de Ingeniería Civil y Mecánica, Universidad Técnica de Ambato, Av. Los Chasquis y Río Payamino, Ambato, 180207 Ecuador; 6 School of Management, Milan, 20156 Italy; 7 RFF-CMCC EIEE, Milan, 20144 Italy

**Keywords:** Energy and behaviour, Environmental economics, Energy economics

## Abstract

This article provides a combined geospatial artificial intelligence-machine learning, geoAI-ML, agent-based, data-driven, technology-rich, bottom-up approach and datasets for capturing the human dimension in climate-energy-economy models. Seven stages were required to conduct this study and build thirteen datasets to characterise and parametrise geospatial agents in 28 regions, globally. Fundamentally, the methodology starts collecting and handling data, ending with the application of the ModUlar energy system Simulation Environment (MUSE), ResidentiAl Spatially-resolved and temporal-explicit Agents (RASA) model. MUSE-RASA uses AI-ML-based geospatial big data analytics to define eight scenarios to explore long-term transition pathways towards net-zero emission targets by mid-century. The framework and datasets are key for climate-energy-economy models considering consumer behaviour and bounded rationality in more realistic decision-making processes beyond traditional approaches. This approach defines energy economic agents as heterogeneous and diverse entities that evolve in space and time, making decisions under exogenous constraints. This framework is based on the Theory of Bounded Rationality, the Theory of Real Competition, the theoretical foundations of agent-based modelling and the progress on the combination of GIS-ABM.

## Background & Summary

At the basic level of most climate-energy-economy models, a main assumption rules input treatment, calculations, and analysis of results. Millions of consumers are deliberately represented as a single agent that takes prices as given, making rational choices with perfect knowledge of the market under rational expectations to maximize welfare, subject to budget constraints^1^, also called a hyperrational representative agent^2^. To overcome the limitations of representative homogenous hyper-rational agents in traditional climate-energy-economy models – so called *the mainstream* – the representation of the human dimension requires the use of empirical, historical, and analytical data. Geospatial big data analytics (combination of Geographical Information Systems, GIS, and Big Data Analytics) and agent-based modelling (ABM) tools present a potential opportunity to introduce the human dimension into the analysis in a more realistic manner. These tools can capture the complexities of heterogeneous shaping structures and the diverse shaping attributes of agents that evolve in space and time, which are driven by bounded rational expectations and exogenous factors. These complexities do not always allow agents to maximise their decisions, however, complexities representation presents an opportunity of more realistic assessments. *The alternative* and novel approach presented here, to represent energy economic agents that are heterogeneous, diverse, evolve in space and time, and take decisions under exogenous constraints, is based on (i) the Theory of Bounded Rationality initially described by Simon^3,4^, discussed and expanded by Petracca^5^, (ii) the Theory of Real Competition by Shaikh^2^, (iii) the theoretical foundations of agent-based modelling by Lavoie^6^, and (iv) the progress on the combination of GIS-ABM suggested by Crooks, *et al*.^7^.

**Fig. 1:**
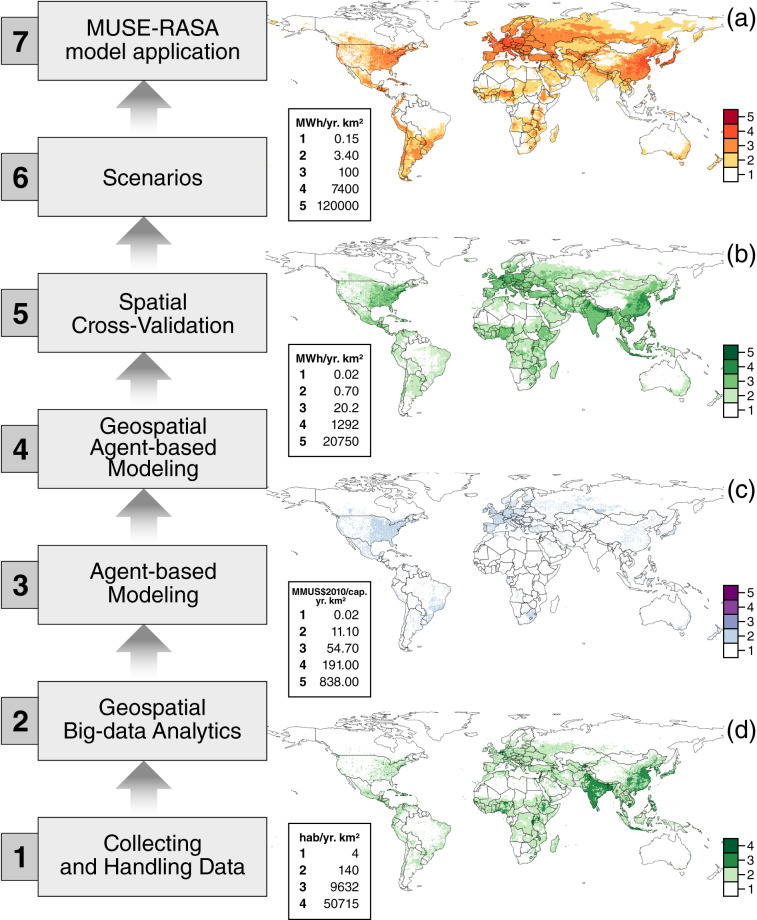
Steps and datasets required to obtain global geospatial agents and energy supply datasets^8^. (**a**) Space heating, SH, (**b**) Space cooling, SC, (**c**) Gross Domestic Product per capita, GDPpc, (**d**) Population count per km^2^. In total, ten global gridded datasets were used in this study. Energy demand datasets with respect to (i) space heating, (ii) water heating, (iii) space cooling, and (iv) total energy demand for heating and cooling, at 1-km^2^ hourly-seasonal resolution, were collected from Sachs, *et al*.^9^. Gridded datasets for (v) heating demand density and (vi) cooling demand density were collected from Sachs, *et al*.^9^. Global socioeconomic and development, and demographic gridded datasets used in this study with respect to (vii) gross domestic product, (viii) gross domestic product per capita, (ix) human development index, and (x) population count per square kilometre were collected from Kummu, *et al*.^10^ and CIESIN^18^.

The following sections provide an account of how the research was conducted, and how the datasets were calculated. Clear and detailed steps were provided for the community to repeat the research and reproduce the results. Details of the available data sources and other previously validated techniques used in this study are also presented here for reference. The datasets collected here are for 2010, because this is the base year used in most models. Figure 1 illustrates the steps of this research, along with some of the datasets required to conduct this research. In step 5, the global geospatial agent dataset is obtained, and from step 7, the energy supply dataset is calculated after applying the MUSE-RASA model^8^. To summarise, this section provides an overview of the datasets required (Subsection 1) for the framework design presented here (Subsection 2).

## Collecting and Handling Data

Spatially resolved and temporally explicit datasets were collected from a range of sources. Missed gridded data were completed where necessary. The five groups of datasets were identified as follows. (i) Gridded end-use energy data were collected for 95 countries and completed for 165 countries. The methodology to complete the missing data and an initial assessment of the gridded dataset was published in Sachs, *et al*.^9^. (ii) Gridded demographic and socioeconomic data were collected from Kummu, *et al*.^10^. (iii) Gridded data for the calibration and validation of energy-related datasets were collected from Department for Business EIS^11^ and ARCONEL^12^. (iv) SSP2 macroeconomic driver data were collected from Riahi, *et al*.^13^. (v) Techno-economic data inputs used in this research is from the MUSE project at Imperial College London’s Sustainable Gas Institute; similar techno-economic data has been used in a series of articles^14–17^. In the following sections, more details on the data used in this study are provided.

### Gridded end-use energy data

Four gridded datasets of the end-use energy of the residential sector were collected from Sachs, *et al*.^9^: (a) space heating, SH; (b) water heating, WH; (c) space cooling, SC; and (d) total energy for heating and cooling, TE. These energy demand datasets had a spatial resolution of 1 km^2^ and hourly seasonal temporal resolution, as explained in Sachs, *et al*.^9^. Figure 1 summarizes the end-use energy datasets used in this study. At this point, there is no processing of the energy data and only the collection. In addition to the end-use energy datasets, data representing the energy demand density were collected from Sachs, *et al*.^9^. Heat density is defined as the ratio between the heating demanded by customers and the area of interest, which may be a district, neighbourhood, or city. Similarly, the cooling density is defined as the ratio between the cooling demand of the customers and the area of interest. At this point, there is no processing of the energy density data.

### Gridded socioeconomic and demographic data

Socioeconomic datasets were collected from Kummu, *et al*.^10^ and refer to (a) gross domestic product (GDP) per square kilometre, (b) gross domestic product per capita, GDPpc, per square kilometre, and (c) Human Development Index, HDI, at the city level or most available level. Demographic datasets were collected from CIESIN^18^ and refer to (d) population count per square kilometre and population density per area of availability.

### Gridded data calibration and validation

Because of the extent of this research in terms of the number of countries covered, the main limitation in terms of data calibration and validation is the requirement for large-scale datasets at high spatiotemporal resolution. To address this limitation, data for validation purposes were collected from two counties: the United Kingdom (UK) and Ecuador. The Department for Business EIS^11^ from UK and ARCONEL^12^ from Ecuador provide publicly available data that were used to validate the gridded energy datasets. The validation process is presented in the validation section of Sachs, *et al*.^9^ for the UK and in Moya, *et al*.^19^ for Ecuador.

### SSP2 macroeconomic drivers

The Shared Socioeconomic Pathways (SSPs) macroeconomic driver datasets are quantitative projections of GDP and Population as part of an Integrated Assessment framework^13^ developed at the International Institute for Applied System Analysis (IIASA, Austria), with a range of other research institutions globally. SSPs have been widely adopted by the climate change research community to analyse the consequences of future climate change. O’Neill, *et al*.^20^ and Van Vuuren, *et al*.^21^ report each of the five scenario narratives and the framework behind each scenario. The matrix used to build the framework combines climate forcing and socioeconomic conditions to describe the situation and evaluate climate impacts, vulnerabilities, adaptation, and mitigation. This research uses the SSP2 scenario datasets for GDP and Population, which is considered a “middle of the road” world, where medium challenges to mitigation and adaptation are assumed^22^. In the SSP2 scenario, trends in social, economic, and technological development broadly follow their historical patterns^23^. Although some countries would make relatively good progress (in the Global North), others would fall short of expectations (in the Global South). Thus, global inequality persists today in terms of development and income growth, and global population growth is moderate^24^. This scenario assumes that governments and civil society will work slowly to achieve sustainable development goals. Overall, a decline in the intensity of resource and energy use is expected; however, environmental systems would experience degradation^25^. SSP2 serves as a starting point to identify the evolution of population and GDP growth in the countries studied in this research.

### Technoeconomic data

The technoeconomic dataset refers to the data used for the economic feasibility analysis of technologies in each region of the world. The economic feasibility analysis is a key study for selecting the most appropriate technology from a set of options. These data were developed by Imperial College London’s Sustainable Gas Institute for the MUSE research project^15–17^. Table 1 provides an example of the technoeconomic data used in the MUSE-RASA model for the evaluation of heating technologies. It is also assumed that the interest rate is 10% and that the initial Capital Expenditure (CAPEX) values are in MUS$2010/PJ.

**Table 1:** Example of the technoeconomic data required in this research.

Technology	Region	Year	CAPEX	Fixed costs	Technical Life	Utilization Factor	efficiency	Fuel
Unit	—	Year	MUS$/PJ	MUS/PJ	Years	%	year	—

CAPEX = Initial Capital Expenditure.

**Fig. 2:**
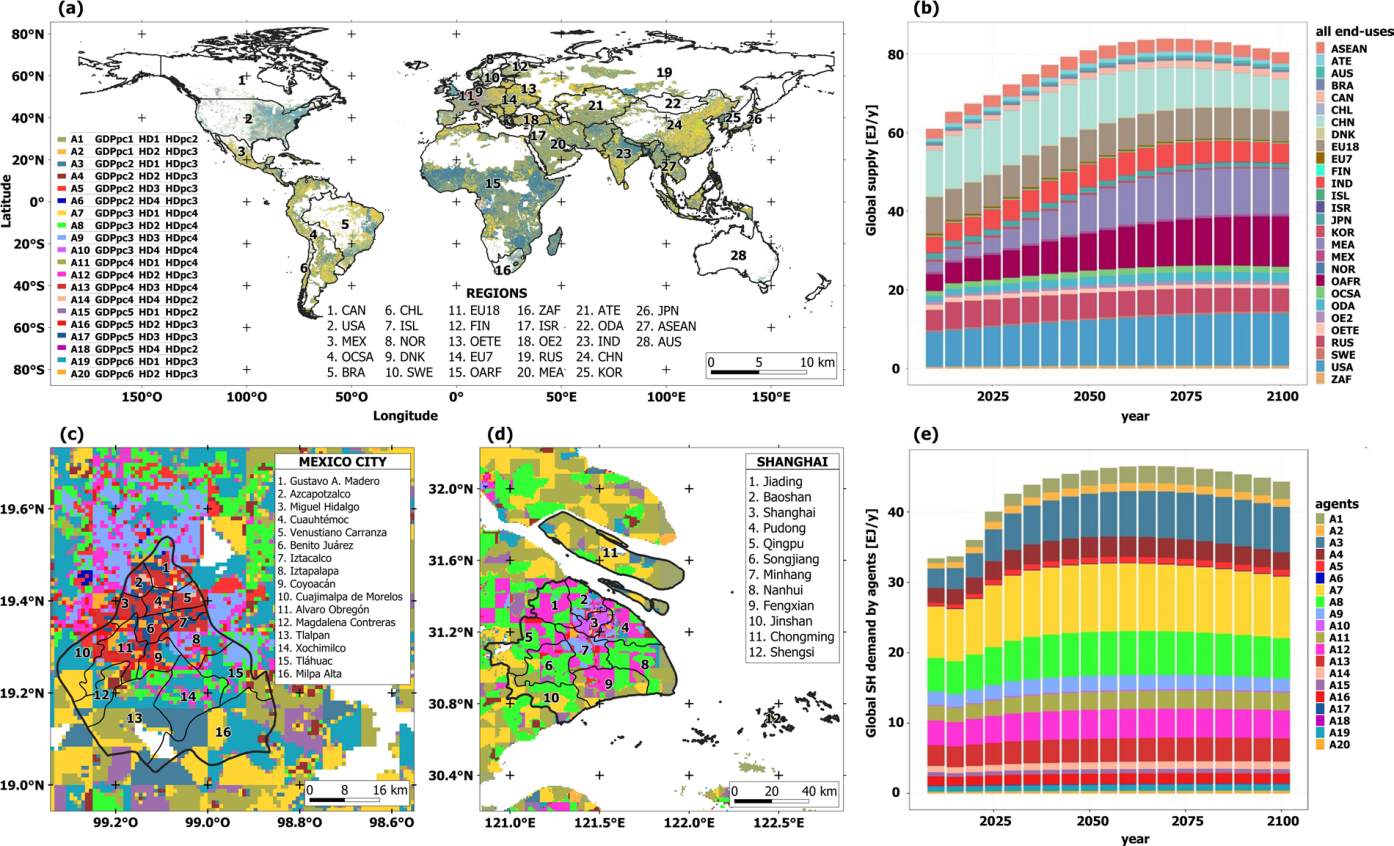
Summary of datasets used and produced in this study (**a**) Global geospatial definition of agent characterisation in terms of three characteristics: GDPpc, HDpc, and HD. (**b**) Global supply of energy in the residential sector by region. (**c**) Geospatial agent distribution in Mexico City. (**d**) Geospatial agent distribution in Shanghai. (**e**) Global supply of heat to the residential sector by agents with three characteristics.

Figure 2 summarises the results of applying the MUSE-RASA framework to obtain the datasets presented herein. A global definition of agent characterisation is provided in terms of GDPpc, HDpc, and HD, as shown in Fig. 2a. Figure 2b presents the global energy demand in the residential sector for the 28 regions in the MUSE-RASA framework. In Fig. 2c,d, a shot of the geospatial agent distribution in Mexico and Shanghai cities is presented. Figure 2e shows the demand for residential heat in terms of the agents’ requirements. These results illustrate the importance of the dataset, along with the strictness and robustness of the systematic approach developed in this study.

## Geospatial Big Data Analytics For Spatial Agent Definition

The geospatial agent-based modelling approach of this study follows five components: (i) agent heterogeneity, (ii) agent diversity, (iii) agent evolution in space and time, (iv) the agent decision-making process, and (v) the influence of exogenous constraints on agent decisions. Geospatial big data analytics, also called spatial data mining, was used to discover hidden knowledge from the large, gridded datasets collected in this research. An Unsupervised Machine Learning technique is applied to classify spatial data points into specific groups according to similar properties with the implementation of the geospatial K-means algorithm developed in this research and published in Sachs, *et al*.^26^. This method has been applied worldwide to the collected datasets.

**Fig. 3:**
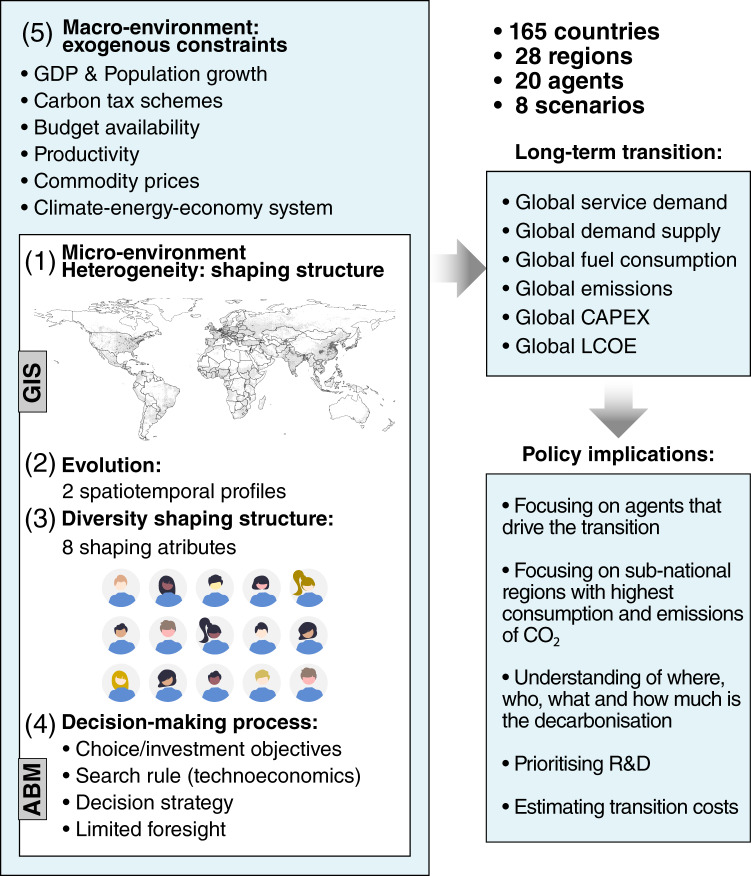
Abstraction from the real world to the MUSE-RASA model, outcomes, and implications. Five components of the geospatial agent-based modelling framework are identified in the micro- and macro-environments of the MUSE-RASA model. The model outcomes and policy implications are also illustrated in the MUSE-RASA environment.

This article aims to introduce a new Geospatial Agent-Based Modelling Framework called MUSE-RASA. The model has been used to create a large dataset of geospatial agents to assess the impact of the climate-energy-economy system on the residential sector globally, with a focus on reaching the mid-century net zero emission (NZE) target. The model uses geospatial big data analytics to capture the human dimension in the modelling approach, which is limited to traditional models. The MUSE-RASA model uses five components–heterogeneity, diversity, evolution, decision-making, and exogenous constraints–to represent the complexities of agents’ structures, diversity, and evolving attributes, as shown in Fig. 3. The model produces global metrics that can be used to analyse transition and design policy recommendations. The MUSE-RASA model is an integrated assessment model that combines GIS-based and ABM approaches and is more realistic in representing the complexities of agent behaviour under different constraints.

## Methods

This research defines an agent as a group of energy consumers with similar characteristics, in terms of heterogeneity, diversity, evolution in space and time, decision-making process and influenced by exogenous constraints. An agent is spatially defined within a specific zone, enclosed by borders under three heterogeneous characteristics. In each of those zones, a range of parameters are calculated to define the agent diversity and evolution. To do this, machine learning, AI-ML-based geospatial big data analytics, a subfield of artificial intelligence (AI), has been systematically applied to a range of datasets. In the following sections, each step of the framework to produce the datasets^8^ shared here is described.

### Spatial agent definition using machine learning

**Fig. 4:**
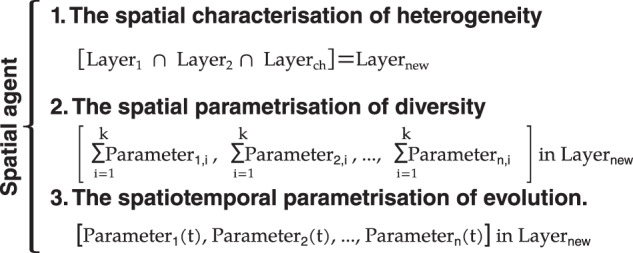
General description of spatial agent definition framework. The heterogeneity, diversity, and evolution of agents are defined using geospatial big-data analytics.

The Spatial Agent Definition consists of three parts: (1) the spatial characterization of heterogeneity, (2) the spatial parametrisation of diversity, and (3) the spatiotemporal parametrisation of evolution. Figure 4 provides a general description of each of the three parts of the spatial agent definition.

This research defines agent heterogeneity as the shaping structure that shapes agent behaviour, which can be historical, social, economic, and cultural structures, according to Schoon and Heckhausen^27^ and Shaikh^2^. Here, agent heterogeneity is captured by overlaying more than one gridded layer, where each layer represents one characteristic (see Fig. 4). The resulting emerging layer from the overlaid process represents the shape structure that defines the limits of contours and zones where agents shape their behaviour. Examples of layers with spatial characteristics that define the agent structure in the energy field include the agent income level, their minimum energy consumption level, and their propensity to consume energy.

Agent diversity is given by a range of parameters that can be calculated in each zone. Overall, the total value of the parameters of interest are extracted from each layer of available gridded data. Examples of attributes that can be used for agent diversity parametrisation in the energy field are the total heating energy demand, total cooling energy demand, and level of development according to HDI, among others. Finally, the spatiotemporal agent evolution is given by a range of parameters that evolve over time for each of the agent zones defined in the spatial characterisation.

The geospatial K-means Unsupervised Machine Learning approach was applied to build the spatial agent definition framework described above as the main contribution of this research. This section provides the general spatial agent definition framework, which can be used to define agents worldwide using geospatial big data analytics. The Framework has six steps: (i) clustering of gridded data, (ii) reclassification of clustered data, (iii) zone definition, (iv) spatial characterisation of agent heterogeneity, (v) spatial parametrisation of agent diversity, and (vi) spatiotemporal parametrisation of agent evolution.

#### Clustering of gridded data

In the geospatial k-means clustering approach, the Elbow Method (EM) was applied to define the optimal number of clusters (ONC), which served to define the optimal number of spatial agents as each cluster turned into a group of people with the same spatial attribute: an agent. EM calculates the Within-Cluster-Sum of Squared Errors (WSS) for different number of clusters k and choose the k for which WSS becomes first starts to diminish. The elbow was visible in the plot of WSS versus k. Table 2 defines the steps of the Algorithm of the Elbow method, which is used to define the ONC. The within-cluster variance (or the total within-cluster sum of squares, wss), *W*(*C*_*k*_), of a cluster *C*_*k*_ is defined by the Euclidean distance in Eq. 1.

1 $$W\left({C}_{k}\right)=\mathop{\sum }\limits_{{x}_{i}\in {C}_{k}}^{n}{\left\Vert {x}_{i}-{\bar{x}}_{k}\right\Vert }^{2}$$

Where:

- *x*_*i*_ is a data point belonging to the cluster *C*_*k*_
- ${\bar{x}}_{k}$ is the mean value of the points assigned to the cluster *C*_*k*_; also called the cluster centroid, and its values are the coordinate-wise average of the data points in *C*_*k*_.
- {*x*_1_, …, *x*_*n*_ } is the set of observations; they are vectors, with one (longitude, latitude) coordinate per dimension (e.g., gridded HD).

**Table 2:** Algorithm of the Elbow method to define the optimal number of clusters within the K-means approach.

Once the ONC is defined, a global clustering is conducted by the application of the spatial K-means algorithm to the attribute/parameter of interest (e.g., HD), as can be seen in Table 3. The main outcome of this stage is the calculation of elements belonging to a cluster *C*_*k*_, which are defined by lower and upper bounds of each cluster. All the cluster elements are centred around their respective centroids. Then, the lower/upper bounds are defined halfway between each consecutive centroid value. This method defines the limits to which each spatial agent belongs. This was performed for each of the parameters of interest. With one parameter, a spatial agent is defined as an agent with one attribute. In the geospatial agent-based modelling section, more attributes are considered to define the heterogeneous and diverse agents.

**Table 3:** The geospatial K-means (x, y, z) algorithm. .Where x and y represent the longitude and latitude, respectively, and z represents a gridded variable that defines the agents.

#### Reclassification of clustered data

**Fig. 5:**
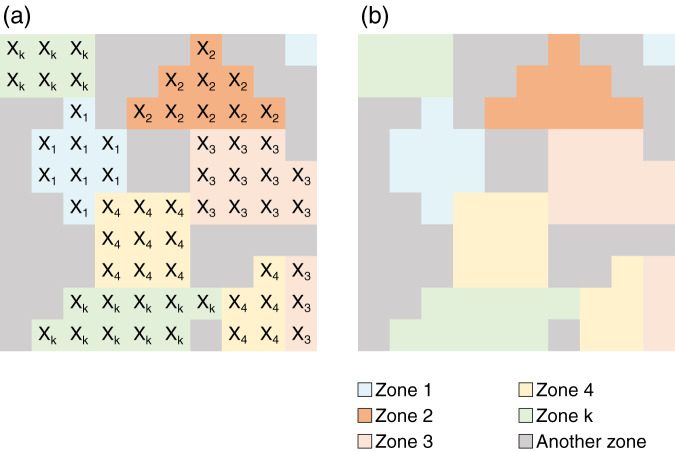
(**a**) Reclassified clustered dataset; (**b**) Geometry of each polygon to define the zone containing the agents. Zone 1 is defined by 2 polygons; Zone 2 is defined by 1 polygon, Zone k is defined by 2 polygons, and Another Zone is defined by 6 remaining polygons.

The global reclassification is done by assigning a number, from 1 to k, to the reclassifying ranges (clustered layer) of values of the gridded dataset. This operation reclassifies groups of values into other values. For example, all values between 1 (lower bound) and 100 (upper bound) become 1 (first segment), and all values between 101 (lower bound) and 200 (upper bound) become 2 (second segment), and so on, until k segments. The lower and upper bounds used to define the reclassification boundaries were obtained in the previous step by using the geospatial k-means clustering algorithm. A reclassified gridded layer is obtained from this step, which is then used to define the zones (in the literature, also known as polygons or areas) where each agent is located. Table 4 presents the general concept of reclassification of gridded data. This also visually explained in Fig. 5a.

**Table 4:** Clustered layer reclassification.

Cluster	Gridded dataset (clusters)				Cluster bounds			Reclass
					lower	upper		
1	*x*_*i*_	*x*_*i*_	*x*_*ii*_	→	*x*_*min*_	*x*_*ii*_	→	*X*_1_
2	*x*_*iii*_	…	*x*_*n*_		_…_	_…_		*X*_2_
k	*x*_*n+1*_	…	*x*_*max*_		*x*_*n+1*_	*x*_*max*_		*X*_*k*_

On the left, all elements of each cluster are identified according to each lower/upper bound. Subsequently, all values belonging to one cluster are assigned a single value. For example, all values from *x*_*min*_ to *x*_*ii*_ of cluster 1 become *X*_1_.

Where:

- *x*_*min*_ is the minimum value in the gridded dataset
- *x*_*max*_ is the maximum value in the gridded dataset
- *x*_*i*_, *x*_*ii*_, *x*_*n*_, *x*_*n+1*_ are the elements of each cluster *C*_*k*_
- $${X}_{i}=\left\{{X}_{i}{\rm{| }}i\in {\mathbb{R}},1\le i\le k\right\}$$
- *X*_*k*_ is the ONC + 1

#### Zones definition

Once the reclassified layer is obtained, the spatial geometry containing the agents within each reclassified cluster is calculated. The spatial geometry is then defined as a zone containing the agents. A zone is defined as a range of finite polygons formed by the contours/boundaries of all contiguous reclassified clusters, as shown in Fig. 5. For example, Zone 1 is defined by two polygons as it is for Zones 3, 4, and k, whereas Zone 2 is defined by a single polygon. Another Zone can be defined by the remaining six polygons, as shown in Fig. 5b.

**Fig. 6:**
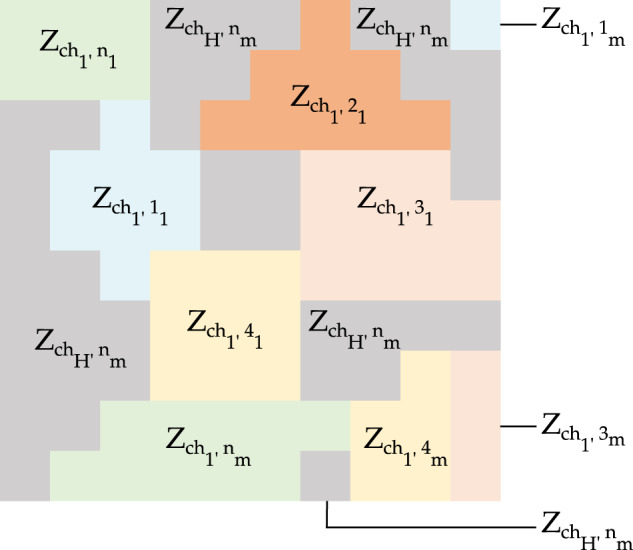
General definition of zones *n* with multiple polygons *m* for one spatial characteristic, ${Z}_{{ch}_{1},{n}_{m}}$. For example, the Zone ${Z}_{{ch}_{1},{2}_{3}}$ represents the zone n = 2 with characteristic 1, *ch*_*1*_, with polygons m = 3.

The general notation used to define a Zone *Z* with one spatial characteristic *ch*_*H*_ is presented in Table 5 and illustrated in Fig. 5. This notation is key for the further definition of agents with multiple characteristics, as developed in the following sections. For example, a spatial agent with 2 spatial characteristics would be defined with the use of two zones each with a different spatial characteristic *ch*_*1*_ and *ch*_*2*_: ${Z}_{c{h}_{1},{n}_{m}}$, and ${Z}_{c{h}_{2},{n}_{m}}$. In Fig. 6, the definition of zones for agents with one spatial characteristic ch_1_ is illustrated. In Table 5, the general notation is also provided for Zones *Z* with 1 to *H* spatial characteristics, 1 to *n* zones, and 1 to *m* polygons.

**Table 5:** Definition of zones with a single spatial characteristic.

Definition	Notation	General notation
Zones n with m polygons and H characteristics ch.	${Z}_{c{h}_{H},{n}_{m}}$	${Z}_{c{h}_{\left(1\to H\right)},zones{\left(1\to n\right)}_{polygon\left(1\to m\right)}}$
Zone 1 with 1 polygon and characteristics 1.	${Z}_{c{h}_{1},{1}_{1}}$	

For example, ${Z}_{{ch}_{2},{3}_{4}}$ represents Zone 3 with 4 polygons and the single characteristic 2. The first characteristic can be HD and the second characteristic can be GDP.

Where:

- *Z* represents a zone, grouping several polygons with similar characteristics to the spatial agent in place. *Z* is defined by a spatial characteristic *ch*, several zones *n*; the grouped polygons m with similar properties forms a zone *Z*.
- *ch* is a spatial characteristic and varies from 1 to H. These can be GDP, GDPpc, and SH, among others.
- *n* is the maximum possible number of zones Z in a region or country.
- *m* is the number of polygons that each zone Z may possess.

#### Spatial characterization of agent heterogeneity

Once the zones were defined, the spatial agent heterogeneity was defined by the spatial characterisation. First, a spatial agent is the join of all zones into a multi-polygon zone with a specific characteristic. Second, a spatial agent with one spatial characteristic defines the heterogeneity with a single characteristic. Table 6 provides the definition of a spatial agent *SpA* with one spatial characteristic *M, ch*_*M*_, in any zone n of a region or country (Eq. 6). It is important to clarify that here, the zone n is already grouped into a single multipolygon. The attribute is a quantity based on annual values, consistent with the selection of agents and the available data. Examples of spatial characteristics that define the agent heterogeneity include energy demand per capita, energy density, and GDP per capita, among others.

**Table 6:** Definition of spatial agents with a single spatial characteristic.

Definition	Equations	
Spatial agent *SpA* with characteristic *H, ch*_*H*_, of all zones 1	$Sp{A}_{c{h}_{H},1}=\mathop{\bigcup }\limits_{i=1}^{m}{Z}_{c{h}_{H},{1}_{i}}={Z}_{c{h}_{H},{1}_{1}}\cup \ldots \cup {Z}_{c{h}_{H},{1}_{m}}$	(Eq. 4)
Spatial agent *SpA* with one characteristic *ch*_1_ in any zone n of a region or country	$Sp{A}_{c{h}_{1},n}=\mathop{\bigcup }\limits_{i=1}^{n}Sp{A}_{c{h}_{1},i}=Sp{A}_{c{h}_{1},{1}_{m}}\cup \ldots \cup Sp{A}_{c{h}_{1},{n}_{m}}$	(Eq. 5)
Spatial agent *SpA* with characteristic *H, ch*_*H*_, in any zone n of a region or country	$Sp{A}_{c{h}_{H},n}=\mathop{\bigcup }\limits_{i=1}^{n}Sp{A}_{c{h}_{H},i}=Sp{A}_{c{h}_{H},{1}_{m}}\cup \ldots \cup Sp{A}_{c{h}_{H},{n}_{m}}$	(Eq. 6)

Spatial agent ${SpA}_{{ch}_{H},1}$ refers to the agent with the attribute H, *ch*_*H*_ defined within the multi-polygon n.

**Fig. 7:**
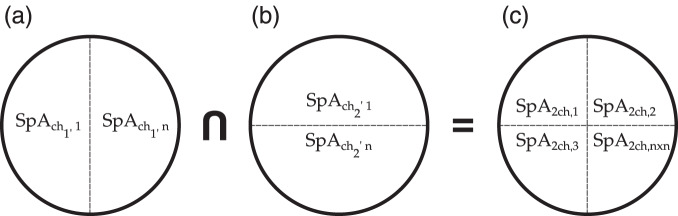
Overlaying calculation for spatially characterised agents with more than one characteristic. Spatial agents with one characteristic and multiple polygons, (**a,b**), are used to generate a new layer (**c**) with a spatial agent with two spatial characteristics and multiple polygons.

The spatial characterisation of agent heterogeneity is given by multiple spatial characteristics. To obtain an agent with multiple spatial characteristics, the spatial characterisation approach for one spatial characteristic is applied to more than one reclassified gridded layers. Then, multiple layers are overlaid to calculate a new layer that intrinsically inherits the heterogeneous characteristics of the layers used for the intersection. For example, from the intersection of two layers (within a range of zones), a new layer that represents new heterogeneous zones emerges. These zones determine the limits or boundaries of agents with similar spatial characteristics and the same number of characteristics as the layers are intercepted. Figure 7 illustrates the process of the overlaying calculation using two spatial agent characteristics separately (a, b) to end with a new emergent agent with two spatial characteristics (c). A multiple spatial characterisation overlays multiple layers to define the agent heterogeneity.

Equation 7 presents the general representation of a spatial agent *SpA* with multiple spatial characteristics *Mch* for a country or a region. The approach used to define spatial agents with multiple spatial characteristics is rooted in the intersection of layers that were previously reclassified using the K-means clustering technique. This definition can be applied to any set of parameters (e.g., GDP, SH, SC, and DH) in the energy field or in any other field where gridded data are available.

7 $$Sp{A}_{Mch}=\mathop{\bigcap }\limits_{j=1}^{M}Sp{A}_{chj}=Sp{A}_{c{h}_{1}}\cap \ldots \cap Sp{A}_{c{h}_{H}}$$

Where:

- *SpA* represents a spatial agent.
- *Mch* defines the multiple spatial characteristics of a spatial agent.
- *ch*_1_ is the first spatial characteristic of the spatial agent.
- *ch*_*H*_ is the spatial characteristic, H, of the spatial agent.

#### Spatial parametrisation of agent diversity

The spatial parametrisation of agent diversity consists of extracting the total value of a parameter or a range of parameters from the multi-polygon zone of each spatial agent. This means that, in each new emergent zone of Fig. 7c, for example, the total value of a parameter is calculated. Table 7 illustrates the equations used to conduct the agent parametrisation of this study with multiple spatial characteristics. The spatial parametrisation can be applied to spatial agents characterised by one or multiple characteristics. A spatial agent *SpA* defined from the intersection of multiple spatial characteristics *Mch* in zone n, *z*_*n*_, with parameter 1, *p*_1_ is defined by $Sp{A}_{Mch,{z}_{n}}\left({p}_{1}\right)=\mathop{\sum }\limits_{i=1}^{k}{p}_{1,i}$, as shown in Table 7.

**Table 7:** Definition of spatial agents with multiple parameters.

Definition	Equations	
Parameter *q, p*_*q*_, in zone n, *z*_*n*_, of spatial agent with multi-attributes	$Sp{A}_{Mch,{z}_{n}}\left({p}_{q}\right)=\mathop{\sum }\limits_{i=1}^{k}{p}_{q,i}$	(Eq. 8)
Spatial agent with multi-attributes and multi-parameters, *p*_1_→*p*_*q*_, in zone *n*, *z*_*n*_.	$Sp{A}_{Mch,{z}_{n}}\left({p}_{1},\ldots ,{p}_{q}\right)=\left\{Sp{A}_{Mch,{z}_{1}}\left({p}_{1}\right),\ldots ,Sp{A}_{Mch,{z}_{n}}\left({p}_{q}\right)\right\}$	(Eq. 9)
Spatial agent with multi-attributes and multi-parameters, *p*_1→_*p*_*q*_, for a country or region with *n* zones, *z*_1→*n*_.	$$Sp{A}_{Mch,{z}_{1\to n}}\left({p}_{1},\ldots ,{p}_{q}\right)=\left\{\begin{array}{ccc}Sp{A}_{Mch,{z}_{1}}\left({p}_{1}\right) & \cdots & Sp{A}_{Mch,{z}_{1}}\left({p}_{q}\right)\\ \vdots & \cdots & \vdots \\ Sp{A}_{Mch,{z}_{n}}\left({p}_{1}\right) & \cdots & Sp{A}_{Mch,{z}_{n}}\left({p}_{q}\right)\end{array}\right\}$$	(Eq. 10)

The parametrisation of a spatial agent *SpA* defined from the intersection of multiple spatial attributes *Mch* in Zone 1, *z*_1_, with one parameter *p*_1_, is given by ${SpA}_{Mch,{z}_{1}}\left({p}_{1}\right)=\mathop{\sum }\limits_{i=1}^{k}{p}_{1,i}$.

#### Spatiotemporal parametrisation of agent evolution

The spatiotemporal parametrisation of agent evolution is given by Eq. 11 and consists of the evolution in time *t* of a parameter or a range of parameters from the multi-polygon zone of each spatial agent. This means that, in each new emergent zone of Fig. 7c, for example, a parameter profile is calculated for a period in time *t*. Equation 11 illustrates the equation used to parametrise the agent evolution with multiple spatial characteristics. The spatiotemporal parametrisation of agent evolution can be applied to spatial agents characterised by one or multiple characteristics.

11 $$Sp{A}_{Mch,{z}_{1\to n}}\left({p}_{1},\ldots ,{p}_{q},t\right)=\left\{\begin{array}{ccc}Sp{A}_{Mch,{z}_{1}}\left({p}_{1},t\right) & \cdots  & Sp{A}_{Mch,{z}_{1}}\left({p}_{q},t\right)\\ \vdots  & \cdots  & \vdots \\ Sp{A}_{Mch,{z}_{n}}\left({p}_{1},t\right) & \cdots  & Sp{A}_{Mch,{z}_{n}}\left({p}_{q},t\right)\end{array}\right\}$$

Where:

- *SpA* represents a spatial agent.
- *Mch* defines the multiple spatial characteristics of a spatial agent.
- *ch* is the spatial characteristic of the spatial agent.
- *z*_1→*n*_ is the zones of the spatial agent.
- *p*_1→*q*_ is the multiple evolving parameters of the agent.
- *t* is the time of the multiple evolving parameters of the agent

### Agent-based modelling

Here, an agent is defined as an autonomous, heterogeneous, diverse, adaptive decision-making entity within a complex system that interacts with its environment and other agents through prescribed conflicting bounded behavioural rules, shaped by shaping structures and attributes, to produce emergent and complex system-level patterns in space and time. To represent this agent definition, this research has proposed the general framework for the spatial agent definition developed here and has adopted the MUSE ABM framework proposed in Giarola, *et al*.^15^, García Kerdan, *et al*.^16^, Moya, *et al*.^17^, and Moya, *et al*.^28^.

#### MUSE ABM framework

**Fig. 8:**
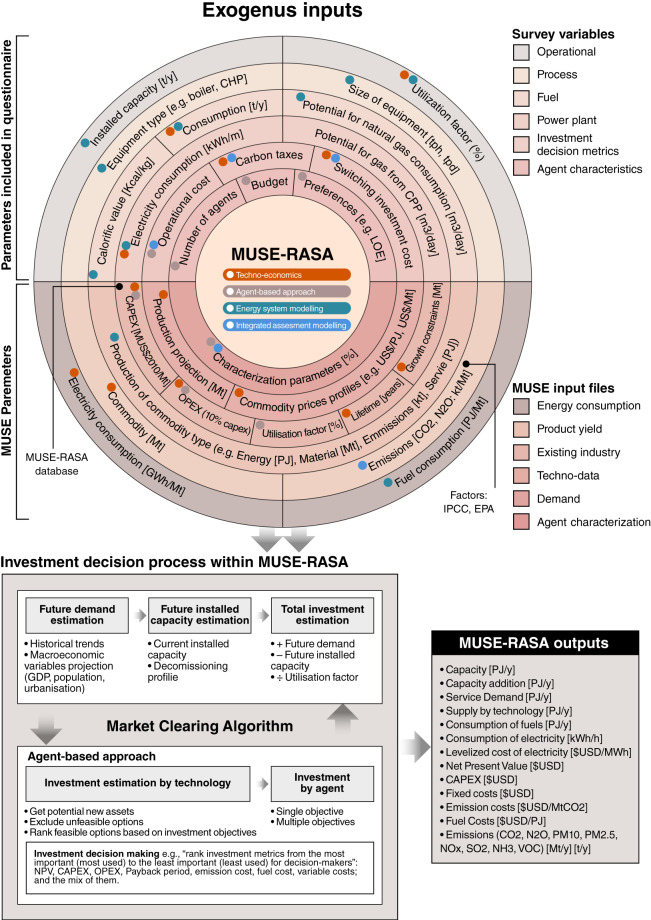
Data flow and MUSE agent-based, bottom-up Integrated Assessment Model that considers the end-use sectors with different levels of detail.

Figure 8 shows the MUSE ABM framework adopted in this study. Exogenous data are required for the model inputs, which are a combination of gridded and national datasets. The MUSE ABM framework defines a decision-making process for each agent based on the 10 parameters listed in Table 8.

**Table 8:** Attribute definition of the agent decision-making process in MUSE^17,37^.

Agent parameters	In Eq. 12	Definition
Objectives	Obj.	A combination of economic, environmental, and technology aspects along with personal motivations.
Search Rule	SR	A collection of information about available technologies and processing abilities of the decision makers. SR leads to the search space (SS) of each agent which includes all defined possible technologies in the energy sector.
Decision strategy	DS	There are two DSs: single- and multi-objective. The single-objective DS uses a merit-order approach where technologies are ranked according to the main agent’s objective. Three possible multi-objective DS approaches are implemented within MUSE^37^.
Type, new or retrofit	TP	Two types of agents: new or retrofit. There is a distinction between retrofit and new equipment.
		It requires a linkage of each new agent to one retrofit agent in order to transfer its stock to a retrofit agent for the later renewal of the assets.
Budget	B	Refers to the maximum budget (expend limit) that each agent can allocate for technology investment.
Maturity Threshold	MT	Indicates the market share that a technology needs to have before it appears in the SS of an agent. This value varies according to agent’s openness towards new technologies.
Technology Stock	TS	A set of technologies available for each agent, obtained via calibration to energy balance and surveyed data.
Technology Ownership	TO	The percentage of each technology that an agent owns in the base year as a result of the calibration.
Agent Population Percentage	PP	Proportion to the total demand based on the percentage of the population represented by agent (obtained from statistics or surveys)
Heat density restriction	HDR	Refers to the technology restriction depending on the actual heat density of the zone that an agent belongs to. This is a particular feature developed and applied for this research into the MUSE framework.

Equation 12 illustrates the agent definition in the MUSE ABM framework. Ten attributes are considered to define the agent decision-making process. The attributes are listed in Table 8.

12 $$A=\left\{Obj,SR,DS,TP,B,MT,TS,TO,PP,HDR\right\}$$

#### Survey-based decision-making parametrisation

This research has also developed three questionaries to collect primary data directly from main sources through *in situ*, person-to-person, and online surveys. The first questionnaire was developed by a team of researchers and industry experts to assess the Indian industry sector; details can be found in Moya, *et al*.^17^. Table 9 expands the use of survey outputs to the MUSE agent decision-making framework. Each parameter of the agent’s definition of Eq. 12 is parametrised by a set of answers from the Questionnaire (see Table 9). For example, in Question 19, the agent is asked about the main investment decision metric to consider when energy technology investment is required. The answer guides the researcher towards the definition of the first parameter of the agent definition, the objective investment. A similar approach was used for the remaining parameters of the agent definition. This questionnaire and survey experience served to further develop a questionnaire for the residential sector in China and Ecuador. The Spanish version of the survey used for the Ecuadorian case study can be found in [https://forms.office.com/r/B93BxJgxX2] and published in Moya, *et al*.^29^ and the Chinese version of the survey can be found in the following link [https://www.wjx.cn/vj/w8Xp3UL.aspx].

**Table 9:** Agent parametrisation of the decision-making process in MUSE based on survey findings.

Agent attribute	In Eq. 12	Agent’s parametrisation based on survey	Survey questions formulation
			Questionnaire in provided links
Objectives	Obj.	Capital expenditure	Question 19
		Operational Cost	
		Net Present Value	
Search Rule	SR	Investors are found to be sophisticated, open to innovations and risk under certain circumstances, and able to gather information on all available natural-gas-based technologies.	Question 10
			Question 13
			Question 15
Decision strategy	DS	Multi-objective. The Weighted Sum is applied which transforms the set of objectives into a single-objective by multiplying each objective with a pre-defined weight.	Question 19
Type, new or retrofit	TP	Both new and retrofit agents are found from the survey.	Question 10
Budget	B	Each enterprise provides their available budget to invest in fuel-switching technologies.	Question 21
			Question 22
			Question 24
Technology Stock	TS	The current technologies in place in addition to natural-gas-based technologies are considered.	Question 13
Agent Population Percentage	PP	The PP is known from the total of surveyed enterprises.	From the total of surveyed enterprises

### Geospatial agent-based modelling framework

**Fig. 9:**
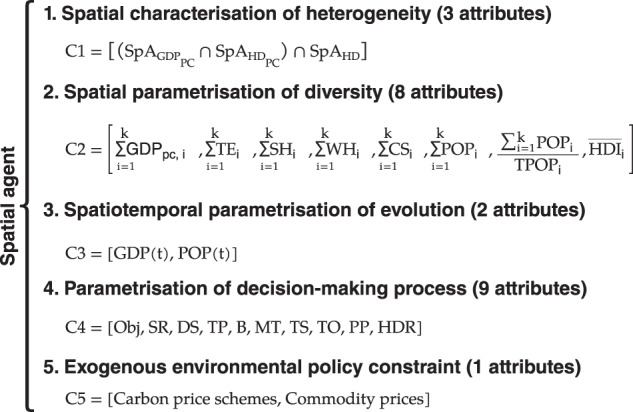
Geospatial Agent-Based Modelling Framework to capture realism in terms of five components: (1) heterogeneity, (2) diversity, (3) evolution, (4) decision-making, and (5) exogenous constraints of multiple agents within climate-energy-economy models. Components (C1, C2, C3, C4, C5); Spatial Agent with GDPpc attribute (${SpA}_{{GDP}_{PC}}$); Spatial Agent with Heat Demand per capita, HDpc, attribute (${SpA}_{{HD}_{PC}}$); Spatial Agent with Heat Density attribute (*SpA*_*HD*_). Aggregated end-use energy demand (TE); aggregated space heating demand (SH); aggregated water heating demand (WH); aggregated space cooling demand (SC); aggregated population (POP); Total population (TPOP); Median Human Development Index ($\overline{HDI}$). Timse (t). Investment objective (Obj); Search rule (SR); Decision strategy (DS); Type, new or retrofit (TP); Budget (B); Maturity threshold (MT); Technology stock (TS); Technology ownership (TO); Population percentage (PP). Carbon Price Scheme (CP). Heat density restriction (HDR).

The components of the geospatial Agent-Based Modelling Framework of this research are characterised and parametrised with five groups of attributes: (1) heterogeneity, (2) diversity, (3) evolution, (4) decision-making, and (5) exogenous constraints. The framework presented in Fig. 9 provides spatially resolved and temporally explicit model agent-based scenarios to assess the long-term sustainable transition of the residential sector globally. This framework captures the human dimension and introduces realism into climate-energy economy models.

#### Spatial characterization of heterogeneity

**Fig. 10:**
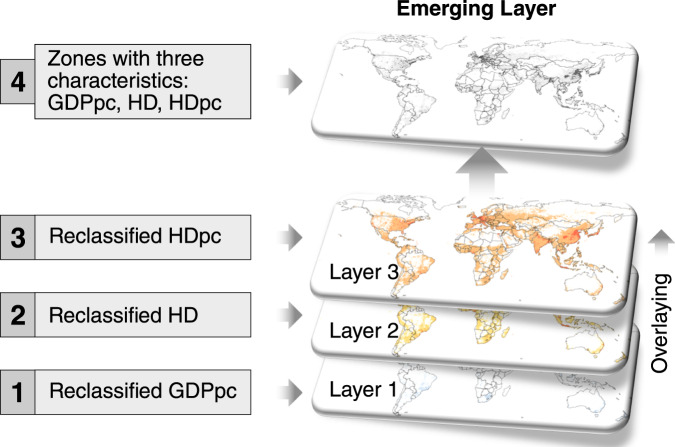
Overlaying calculation to spatially characterised agent heterogeneity with three attributes. Reclassified gridded layers of GDPpc, DH and HDpc are used to produce an emergent layer that captures the shaping structures of agent heterogeneity. From the overlaying emerges a new layer used to estimate the datasets presented in this study^8^.

The spatial characterization of agent heterogeneity follows Step (iv) of the general framework for the spatial agent definition presented previously. The attributes used to define the spatially resolved and time-explicit characteristics are presented in Eq. 13 and are explained in Table 10. Figure 10 illustrates the process of capturing agent heterogeneity by overlaying three shaping structures.

13 $$Emergin\;Layer=\left[\left({SpA}_{{GDP}_{PC}}\cap {SpA}_{HD}\right)\cap {SpA}_{{HD}_{PC}}\right]$$

**Table 10:** Description of the group of attributes (see Fig. 10) for the spatial characterization of agent heterogeneity presented in Fig. 9.

Gridded GDP per capita (GDPpc)	Gross domestic product per capita (GDPpc) is a metric that breaks down a country’s economic output per person and is calculated by dividing the GDP of a country^38^ by its population^39^ in each km^2^.
Gridded heating demand density (HD)	Demand density of heating (HD) in the residential sector globally in each km^2^ is calculated using gridded data from Sachs, *et al*.^9^.
Gridded heat demand per capita (HDpc)	Demand of heating per capita (HDpc) in the residential sector globally at km^2^ spatial resolution is calculated using the framework and heating demand data from Sachs, *et al*.^9^ and population from CIESIN^39^.

#### Spatial parametrisation of diversity

The spatial parametrisation of agent diversity follows the step (v) of the general framework for the spatial agent definition presented here. The attributes used to define the spatially resolved and time-explicit parameters of diversity are presented in Eq. 14, and are explained in Table 11.

14 $$\left[\mathop{\sum }\limits_{i=1}^{k}GD{P}_{i},\mathop{\sum }\limits_{i=1}^{k}T{E}_{i},\mathop{\sum }\limits_{i=1}^{k}S{H}_{i},\mathop{\sum }\limits_{i=1}^{k}W{H}_{i},\mathop{\sum }\limits_{i=1}^{k}S{C}_{i},\mathop{\sum }\limits_{i=1}^{k}PO{P}_{i},\frac{{\sum }_{i=1}^{k}PO{P}_{i}}{TPOP},{\overline{HDI}}_{i}\right]$$

**Table 11:** Description of the group of attributes of component 2 (C2, see Fig. 9) for the spatial parametrisation of agent presented in Eq. 14.

$\mathop{\sum }\limits_{{\rm{i}}=1}^{{\rm{k}}}{{\rm{GDP}}}_{{\rm{i}}}$	Aggregate GDP	Total aggregate gross domestic product in each 3-attribute characterised agent zone represents the total agent economic output per zone. 1-km^2^ resolution gridded data from Kummu, *et al*.^38^ is used.
$\mathop{\sum }\limits_{{\rm{i}}=1}^{{\rm{k}}}{{\rm{TE}}}_{{\rm{i}}}$	Aggregate TE	Total aggregate end-use energy demand in each 3-attribute characterised agent zone represents the total agent energy service demand per zone. 1-km^2^ resolution gridded data from Sachs, *et al*.^9^ is used.
$\mathop{\sum }\limits_{{\rm{i}}=1}^{{\rm{k}}}{{\rm{SH}}}_{{\rm{i}}}$	Aggregate SH	Total aggregate space heating demand in each 3-attribute characterised agent zone represents the total agent space heating demand per zone. 1-km^2^ resolution gridded data from Sachs, *et al*.^9^ is used.
$\mathop{\sum }\limits_{{\rm{i}}=1}^{{\rm{k}}}{{\rm{WH}}}_{{\rm{i}}}$	Aggregate WH	Total aggregate water heating demand in each 3-attribute characterised agent zone represents the total agent water heating demand per zone. 1-km^2^ resolution gridded data from Sachs, *et al*.^9^ is used.
$\mathop{\sum }\limits_{{\rm{i}}=1}^{{\rm{k}}}{{\rm{SC}}}_{{\rm{i}}}$	Aggregate SC	Total aggregate space cooling demand in each 3-attribute characterised agent zone represents the total agent space cooling demand per zone. 1-km^2^ resolution gridded data from Sachs, *et al*.^9^ is used.
$\mathop{\sum }\limits_{{\rm{i}}=1}^{{\rm{k}}}{{\rm{POP}}}_{{\rm{i}}}$	Aggregate POP	Total aggregate population in each 3-attribute characterised agent zone represents the total population belonging to the agent zone. 1-km^2^ resolution gridded data from CIESIN^39^ is used.
$\frac{{\sum }_{{\rm{i}}=1}^{{\rm{k}}}{{\rm{POP}}}_{{\rm{i}}}}{{\rm{TPOP}}}$	POP share	In a given region or country, the POP share is calculated dividing the total aggregated population of a 3-attribute characterised agent zone by the total population of the region or country.
${\overline{{\rm{HDI}}}}_{{\rm{i}}}$	Median HDI	Median Human Development Index in each 3-attribute characterised agent zone represents the total agent degree of development in terms of level of education, access to health services and income level. 1-km^2^ resolution gridded data from Kummu, *et al*.^38^ is used.

Each attribute is calculated by extracting the total aggregate value in each 3-attribute characterised agent zone. k refers to the numbers of elements or data points that belong to each agent zone.

#### Spatiotemporal parametrisation of evolution

The spatiotemporal parametrisation of agent evolution follows the step (vi) of the general framework for the spatial agent definition presented here. The evolving attributes used to define the spatially resolved and time-explicit parameters of agent evolution are presented in Eq. 15.

15 $$C3=\left[GDP\left(t\right),POP\left(t\right)\right]$$

Where:

- *POP*(*t*) is the Population evolution in time
- *GDP*(*t*) in the GDP evolution in time

#### Parametrisation of decision-making process

This study adopted the decision-making process approach of the MUSE ABM framework described in Eq. 12 and are explained in Table 9.

#### Exogenous environmental policy constraint

The external limitations imposed by environmental policies are referred to as exogenous constraints, which can prompt individuals to alter their actions while evaluating heating or cooling technology. To investigate this, the study utilized carbon price profiles from 2005 to 2100 suggested in the MUSE model^30^, with each individual having access to various technologies that could result in varying levels of CO_2_ emissions. The total cost of carbon is calculated when an individual selects a technology that satisfies its service requirements. This external influence affects the decision-making process of each individual before making the ultimate investment decision.

#### Scenario definition

In this study, eight scenarios have been developed (see Table 12) to assess each of the five components of the geospatial Agent-Based Modelling Framework presented previously. Heterogeneity (i), diversity (ii), and evolution (iii) follow the definitions previously discussed. For the decision-making component (iv), this research has adopted the Levelised Cost of Energy (LCOE) as the main investment objective in agents when choosing a technology. The calculation of the annual LCOE for each technology includes the required investment expenditures (including financing), the operations and maintenance expenditures, the fuel expenditures, the electricity generation, the discount rate, and the technical life of the system. It is assumed that the agents would consider the final LCOE value to make the final decision. For the same decision-making component (iv), scenarios are defined assuming that agents have unlimited budgets (scenarios 01, 02, 05, and 06) and that agents have budget restrictions (scenarios 03, 04, 07, 08) according to their GDPpc shaping structure, which is part of the heterogeneity characterisation. The latter is called the multiple-budget system. The heat density restriction (HDR) is added to the decision-making process. HDR defines the technical and economic feasibility of technologies in agent zones according to the heat density of the zone where the agents are located. For the component of the exogenous environmental policy constraint (v), it is assumed that scenarios 01, 03, 05, and 07 consider carbon price (CP) schemes from Budinis, *et al*.^30^. The remaining scenarios do not consider CP schemes in the model.

**Table 12:** Scenario definition based on the five components of the geospatial Agent-Based Modelling Framework.

Scenario	Heterogeneity	Diversity	Evolution	Decision-making process			Exogenous env. policy constraints
				Obj.	Budget	HDR	CP
01	3 att.	8 att.	2 att.	LCOE	Unlimited	Without	With
02	3 att.	8 att.	2 att.	LCOE	Unlimited	Without	Without
03	3 att.	8 att.	2 att.	LCOE	Multiple	Without	With
04	3 att.	8 att.	2 att.	LCOE	Multiple	Without	Without
05	3 att.	8 att.	2 att.	LCOE	Unlimited	With	With
06	3 att.	8 att.	2 att.	LCOE	Unlimited	With	Without
07	3 att.	8 att.	2 att.	LCOE	Multiple	With	With
08	3 att.	8 att.	2 att.	LCOE	Multiple	With	Without

Unlimited budget refers to an agent with infinite budget. Multiple refers to a multi-budget system that is simulated according to agent GDPpc attribute (att.). HDR refers to the heat density restriction of each agent and is associated with the HD attribute of the agent.

### The MUSE-RASA model

**Fig. 11:**
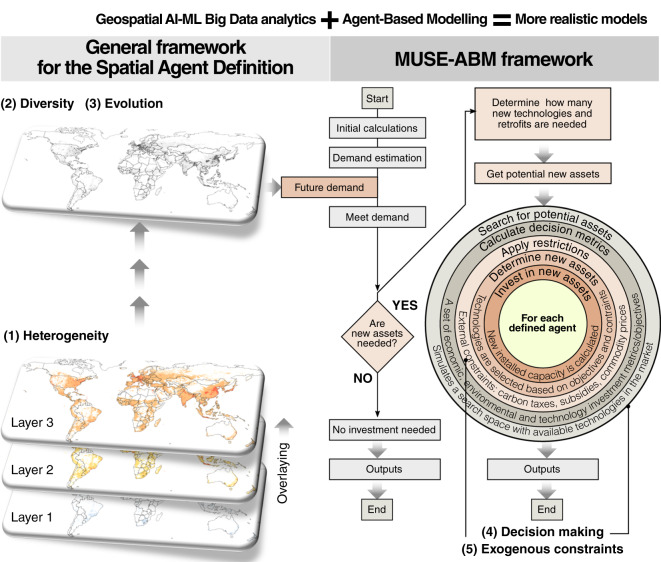
Components of the MUSE (ModUlar energy system Simulation Environment) ResidentiAl Spatial Agent (RASA), MUSE-RASA model. The algorithm of agent-based modelling is presented along with a combination of spatially resolved and time-explicit agents globally. This approach captures the heterogeneity, diversity, evolution, decision-making process, and exogenous constraints of each agent defined in this study.

The MUSE-RASA model is a combination of the general framework for the spatial agent definition and the MUSE ABM Framework used for the geospatial Agent-Based Modelling Framework explained in previous section. Figure 11 presents the link between spatially resolved and time-explicit agents with the MUSE ABM algorithm that has been applied in the MUSE-RASA model. The five components that capture realism in the geospatial Agent-Based Modelling Framework explained previously are also illustrated: (1) heterogeneity, (2) diversity, (3) evolution, (4) decision-making, and (5) exogenous constraints of multiple agents within the MUSE-RASA model. The model calculates six outputs of the eight agent-based scenarios to explore the long-term climate-energy-economy transition pathways towards the NZE targets by mid-century, with a focus on the residential sector globally.

Table 13 describes the formulas that have been implemented in the MUSE-RASA model to calculate the outputs of the model. The service demand for space heating (and other residential end-uses) is firstly calculated. This serves to calculate the installed capacity required to meet the demand for heating supply technologies. Once the technologies are identified, electricity and fuel consumption can be estimated. The total capital expenditure (CAPEX), along with the LCOE and the total emissions are finally calculated.

**Table 13:** Formula implementation and variable description of the MUSE-RASA calculations of metrics that serve to evaluate the long-term transition of the climate-energy-economy system of this research, with a focus on the residential sector.

MUSE-RASA calculations	Formula implementation
Service demand	$\mathop{\sum }\limits_{i=1}^{na}{\left.\left(\frac{(1+w\ast {t}^{n})}{(1+{t}^{n})}\right)\ast \left(\frac{a\ast (1+{t}^{n})}{(1+b{e}^{GDP\ast c})}\right)\right|}_{SA{g}_{na}}$
Supply	$\mathop{\sum }\limits_{i=1}^{na}{(Ins{t}_{cap}\ast {\rm{UF}}\ast {E}_{out})| }_{SA{g}_{na}}$
Fuel/electricity consumption	$\mathop{\sum }\limits_{i=1}^{na}{(Ins{t}_{cap}\ast {\rm{UF}}\ast {E}_{in})| }_{SA{g}_{na}}$
CAPEX	$\mathop{\sum }\limits_{i=1}^{na}{\left.\left({{\rm{TC}}}_{reg}\ast {\left(\frac{Ins{t}_{cap}}{Re{f}_{cap}}\right)}^{tce}\right)\right|}_{SA{g}_{na}}$
LCOE	$\mathop{\sum }\limits_{i=1}^{na}{\left.\left(\frac{{\sum }_{t=1}^{n}\frac{{I}_{t}+{M}_{t}+{F}_{t}}{{\left(1+r\right)}^{t}}}{{\sum }_{t=1}^{n}\frac{{E}_{t}}{{\left(1+r\right)}^{t}}}\right)\right|}_{SA{g}_{na}}$
Emissions	$\mathop{\sum }\limits_{i=1}^{na}{({\rm{FC}}\ast {\rm{ef}})| }_{SA{g}_{na}}$

*SAg*: Spatial agent, *na*: number of agents per region. *w*: weights; *t*: foresight time (5 years is assumed); *a*: 1e6*constant*population; *n*: 4; *b*: constant; *GDP*: Gross Domestic Product; *c*: constant; *Inst*_*cap*_: Installed capacity; *UF*: Utilisation factor; *E*_*out*_: Energy out of the technology supply; *E*_*in*_: Energy into the technology supply; *TC*: Technology cost; *reg*: region; *Ref*_*cap*_: Reference capacity in base year; *tce*: technology scaling capacity exponent; *I*_*t*_: Investment expenditures in year t (including financing); *M*_*t*_: Operations and maintenance expenditures in year t; *F*_*t*_: Fuel expenditures in year t; *E*_*t*_: Energy generation in year t; *r*: Discount rate; *n*: Life of the system; *FC*: fuel consumption; *ef*: emission factor.

## Data Records

The MUSE-RASA geospatial agent-based modelling framework presents 13 geospatial datasets^8^: three for the characterization, two for heterogeneity definition, one for diversity parameterization, one for evolution parameterization, two for decision-making parameterization, one for the estimation of global energy demand in the residential sector, two for spatial cross-validation, and one for the MUSE regions used in this research. Details are presented in Table 14. This research defines characterisation as the process of assigning geospatial boundaries to agents under similar geospatial characteristics and parametrisation as the process of estimating numeric parameters to those agents within those boundaries. This study includes a survey-based decision-making parametrisation for China and Ecuador in Dataset 8. To validate the approach, this study employed the spatial cross-validation technique explained in the methodology section. Overall, this study contributes to a better understanding of complex agent systems and provides insights into how to use data in a spatial context for human representation in models.

**Table 14:** Geospatial datasets provided in this article and their names in the repository^8^.

Geospatial dataset	Name in repository	Format
**To define agent characterisation:**		
1. Global clustered GDPpc	GDPpc_km2_shapes	Shape [.shp]
2. Global clustered HD	HD_km2_shapes	Shape [.shp]
3. Global clustered HDpc	HDpc_km2_shapes	Shape [.shp]
**To define agent heterogeneity:**		
4. Global agents with two characteristics	Agents_GDPpc_HDpc	Shape [.shp]
5. Global agents with three characteristics	Agents_GDPpc_HDpc_HD	Shape [.shp]
6. Dataset to define agent diversity	Global_agents_diversity	Text [.csv]
7. Dataset to define agent evolution in space and time	Global_agents_evolution	Text [.csv]
8. Dataset to define decision-making process in China	China_dm_agents_survey	Text [.csv]
9. Dataset to define decision-making process in Ecuador	Ecuador_dm_agents_survey	Text [.csv]
10. Dataset of global energy demand by agents and regions	Global_agents_demand	Text [.csv]
11. Dataset of global geospatial cross validation	Spatial_cross_validation	Text [.csv]
12. Dataset of global geospatial cross validation errors	Spatial_cross_validation_errors	Text [.csv]
13. Dataset of global MUSE region shapes	Regions_shapes	Shape [.shp]

Viewing or using shape files [.shp] requires GIS software, such as the open-source QGIS application or R geospatial packages.

### Global clustered GDPpc [GDPpc_km2_shapes.shp]

**Fig. 12:**
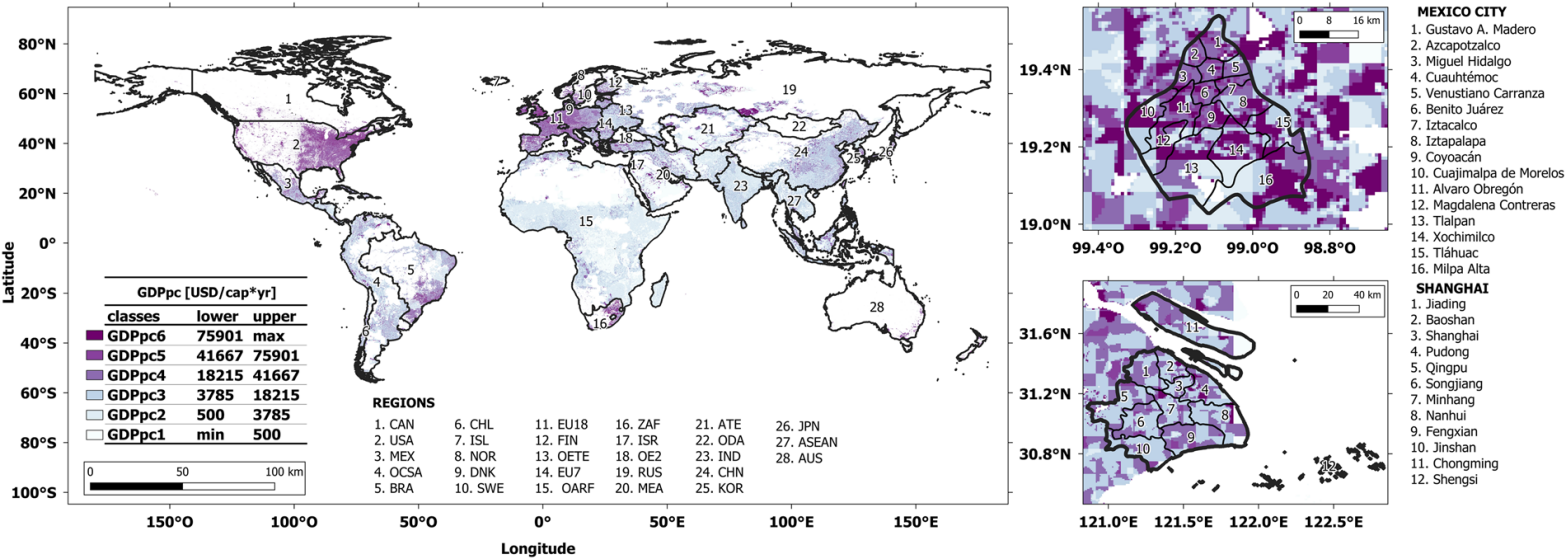
Global geospatial distribution of optimal number of GDPpc-based agent classes. The extreme classes (GDPpc1 and GDPpc6) are defined based on the literature and the remining four classes are the result of a K-means clustering approach, published in Sachs, *et al*.^9^ and Moya, *et al*.^28^. Gridded global datasets for Gross Domestic Product and Human Development Index is used from Kummu, *et al*.^10^. Upper and lower classes for the GDPpc are taken from Stierli^40^. Gridded population counts are taken from CIESIN^18^.

This dataset provides a globally clustered GDPpc with respect to the six classes, as shown in Fig. 12 and Table 15. The shape file presents a range of zones with clustered values, regardless of the geographical administrative areas. For example, agents living in zones within GDPpc limit 1 (GDPpc1 = [min, 500], USD/cap*yr) can be in more than one region.

**Table 15:** GDPpc-based agent classes.

	GDPpc [USD/cap*yr]	
	Lower bound	Upper bound
GDPpc1	min	500
GDPpc2	500	3785
GDPpc3	3785	18125
GDPpc4	18125	41667
GDPpc5	41667	75901
GDPpc6	75901	max

The extreme classes (GDPpc1 and GDPpc6) are defined based on the literature, and the four remaining classes are the result of a K-means clustering approach, published in Sachs, *et al*.^9^ and Moya, *et al*.^28^. Gridded global datasets for Gross Domestic Product were used from Kummu, *et al*.^10^. The upper and lower classes for the GDPpc were obtained from Stierli^40^.

### Global clustered HD [HD_km2_shapes.shp]

This dataset provides a globally clustered heat density with respect to the four classes, as shown in Table 16. The shape file presents a range of zones with clustered values, regardless of the geographical administrative areas. For example, agents living in zones within HD limit 2 (HD2 = [1790, 12080], MWh/km^2^*yr) can be in more than one region.

**Table 16:** Estimated heat density classes based on previously clustered heat density data are explained and published in Sachs, *et al*.^9^.

	Heat density [MWh/km^2^*yr]	
	Lower bound	Upper bound
HD1	min	1790
HD2	1790	12080
HD3	12080	36930
HD4	36930	max

### Global clustered HDpc [HDpc_km2_shapes.shp]

**Fig. 13:**
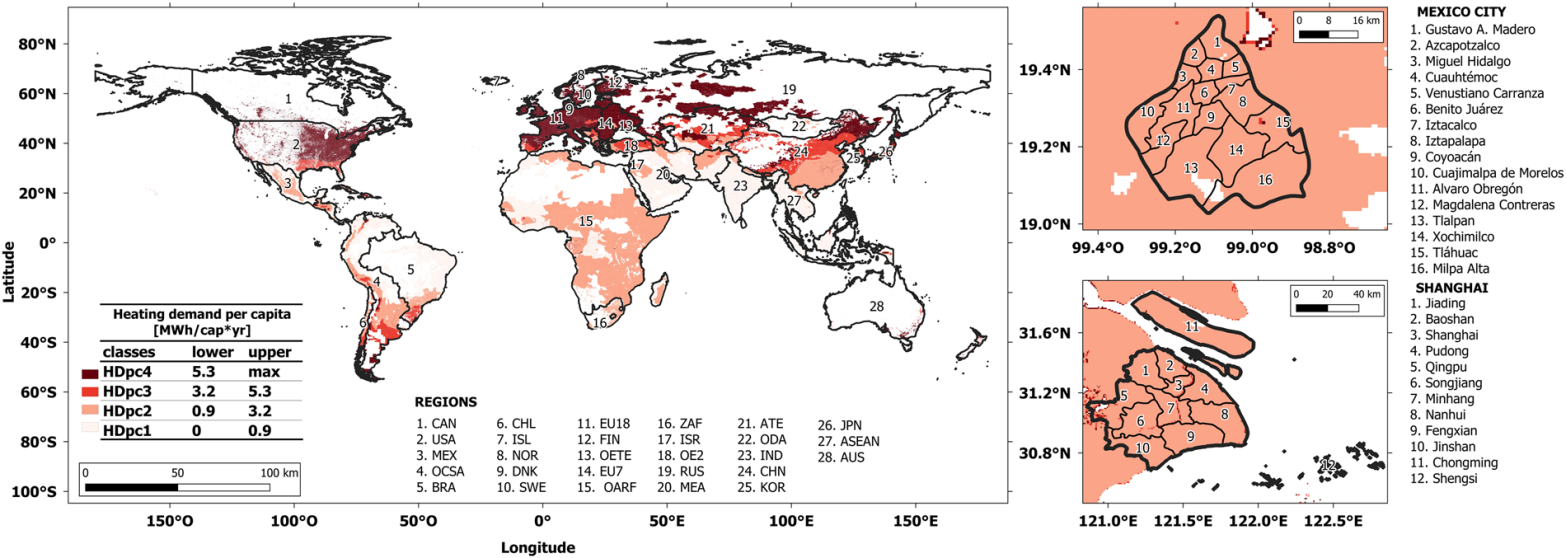
Global geospatial distribution of heat demand per capita. Heat demand gridded data has been collected from Sachs, *et al*.^9^ and Moya, *et al*.^28^. Gridded population counts are taken from CIESIN^18^.

This dataset provides global clustered heat demand per capita with respect to the four classes, as shown in Fig. 13 and Table 17. The shape file presents a range of zones with clustered values, regardless of the geographical administrative areas. For example, agents living in zones within HDpc limit 3 (HD3 = [3.2, 5.3], MWh/cap*yr) can be in more than one region.

**Table 17:** Estimated annual HDpc classes based on literature^41^.

	Heating demand per capita [MWh/cap*yr]	
	Lower bound	Upper bound
HDpc1	min	0.9
HDpc2	0.9	3.2
HDpc3	3.2	5.3
HDpc4	5.3	max

The annual threshold of HDpc is defined globally.

### Global agents with two characteristics [Agents_GDPpc_HDpc.shp]

**Fig. 14:**
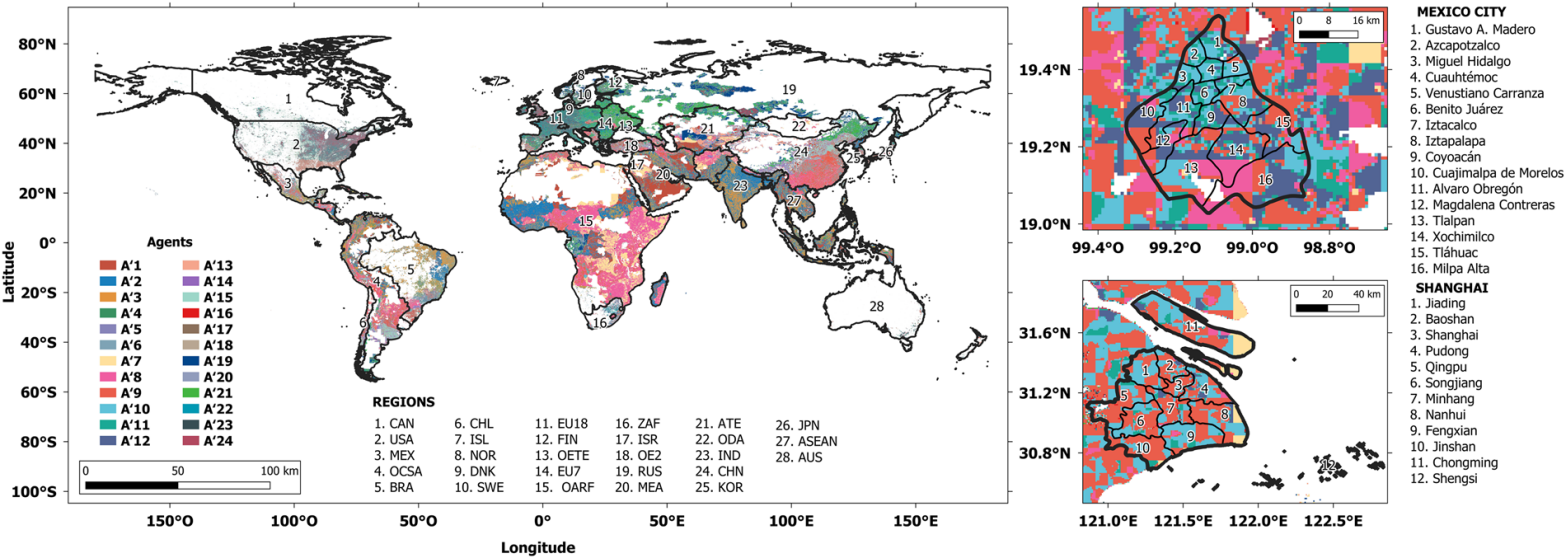
Geospatial representation and distribution of agents with two reclassified attributes: GDPpc and HDpc. For these agents there is no need to conduct a subclustering approach as the maximum number of agents emerge from the combination of 6-GDPpc classes and 4-HDpc classes.

This dataset provides global agent characterisation based on two geospatial characteristics, as shown in Fig. 14 and Table 18. The shape file presents a range of zones that represent the borders or areas where agents with two characteristics interact regardless of geographical administrative areas. For example, agents living in zone A’ 1 belong to areas with GDPpc1 and HDpc1 and are in more than one region globally.

**Table 18:** Global disaggregation for agents defined with two spatial characteristics.

Global agents	Classes		Global agents	Classes	
	[spatially-resolved and time-explicit]			[spatially-resolved and time-explicit]	
	GDPpc	HDpc		GDPpc	HDpc
A’1	1	1	A’13	1	3
A’2	2	1	A’14	2	3
A’3	3	1	A’15	3	3
A’4	4	1	A’16	4	3
A’5	5	1	A’17	5	3
A’6	6	1	A’18	6	3
A’7	1	2	A’19	1	4
A’8	2	2	A’20	2	4
A’9	3	2	A’21	3	4
A’10	4	2	A’22	4	4
A’11	5	2	A’23	5	4
A’12	6	2	A’24	6	4

GDPpc: Gross Domestic Product per capita; HDpc: Heat Demand per capita; A’: agent with 2 spatial characteristics. Refer to the previous tables for the class values. e.g., Agent’ 1 (A’1) belongs to zones, anywhere in the world, with a gridded GDPpc up to 500 US$/y (GDPpc = 1), and a gridded HDpc up to 3.2 MWh/y*cap (HDpc = 2).

### Global agents with three characteristics [Agents_GDPpc_HDpc_HD.shp]

This dataset provides global agent characterisation based on three geospatial characteristics, as shown in Fig. 2 and Table 19. The shape file presents a range of zones that represent the borders or areas where agents with the three characteristics interact, regardless of geographical administrative areas. For example, agents living in zone A2 belong to areas with GDPpc1, HDpc2 and HD1, and are in more than one region globally.

**Table 19:** Global disaggregation for agents defined with three spatial characteristics.

Global agents	Classes			Global agents	Classes		
	[spatially-resolved and time-explicit]				[spatially-resolved and time-explicit]		
	GDPpc	HDpc	HD		GDPpc	HDpc	HD
A1	1	2	1	A11	4	4	1
A2	1	3	2	A12	4	3	2
A3	2	3	1	A13	4	4	3
A4	2	3	2	A14	4	2	4
A5	2	3	3	A15	5	2	1
A6	2	3	4	A16	5	3	2
A7	3	4	1	A17	5	3	3
A8	3	4	2	A18	5	2	4
A9	3	4	3	A19	6	3	1
A10	3	4	4	A20	6	3	2

GDPpc: Gross Domestic Product per capita; HD: Heat Density; HDpc: Heat Demand per capita; A: agent. Refer to the previous tables for the class values. e.g., Agent 1 (A1) belongs to zones, anywhere in the world, with a gridded GDPpc up to 500 US$/y (GDPpc = 1), a gridded HD up to 1790 MWh/km^2^ (HD = 1), and a gridded HDpc up to 3.2 MWh/y*cap (HDpc = 2).

### Dataset to define agent diversity [6_global_agents_diversity.csv]

**Fig. 15:**
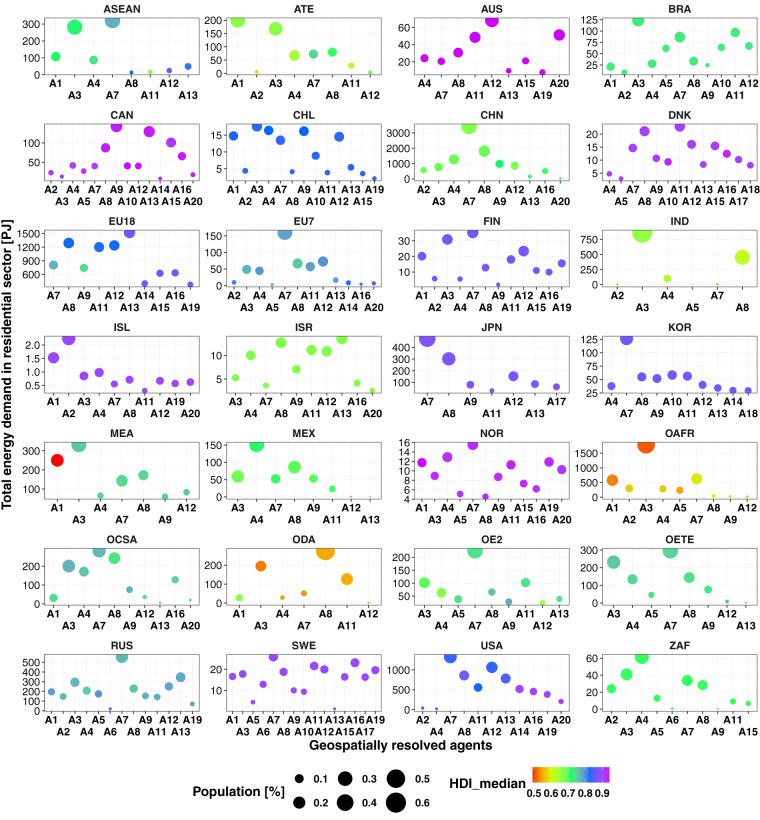
Region-based disaggregation of Total Residential Energy Demand, Human Development Index and population share for the geospatial parametrisation of agent diversity.

This dataset provides 12 parameters to define agent diversity worldwide aggregated in 28 regions. All the values were provided in 2010. Table 20 defines each variable provided in this dataset. Figure 15 provides the global distribution of three out of twelve parameters that define the agent diversity for each of the 28 regions considered in this research.

**Table 20:** Definition of variables presented in dataset 6.

Variable	Definition	Variable	Definition
area_km2	Inhabited area covered by each agent	POP_sum	Total population in each agent zone per region
GDP_sum.USMM	Total GDP per agent in US$ million	Pop_perc	Shared population in each agent zone per region
ET_sum_PJ	Total energy demand in the residential sector by agent	HDI_med	Median human development index in each agent zone per region
SH_sum	Total space heating demand in the residential sector by agent	GDPppZones_2clustering	GDPpc geospatial agent characteristic after subclustering
WH_sum	Total water heating demand in the residential sector by agent	HDpcZones_2clustering	HDpc geospatial agent characteristic after subclustering
SC_sum	Total space cooling demand in the residential sector by agent	HDZones_2clustering	HD geospatial agent characteristic after subclustering

Twelve parameters are provided to define agent diversity for 28 regions worldwide.

### Dataset to define agent evolution in space and time [7_global_agents_evolution.csv]

This dataset provides the values of GDPpc and Population for each agent zone in each region from 2010 to 2100.

### Dataset to define the decision-making process in China [8_China_dm_agents_survey.csv]

This dataset provides a range of variables to define the current status of the residential sector in China in terms of energy consumption and willingness to invest in new energy technologies or retrofitting. Variables are self-explanatory.

### Dataset to define the decision-making process in Ecuador [9_Ecuador_dm_agents_survey.csv]

This dataset provides a range of variables to define the current status of the residential sector in Ecuador, in terms of energy consumption and willingness to invest in new energy technologies or retrofitting. Variables are self-explanatory.

### Dataset of global energy demand by agents and regions [10_global_agents_demand.csv]

This dataset provides the energy demand in the residential sector worldwide, disaggregated by agents and regions. The demand is further dissagregated in six service demands, as follows: space heating (hspace), water heating (hwater), space cooling (cspace), cooking (cook), lighting (light) and appliances (appl). These demands were used in eight previously defined scenarios. Figure 2 illustrates this dataset.

### Dataset of global geospatial cross-validation [11_spatial_cross_validation.csv]

This dataset provides details of the results of the subclustering approach used in this study. The subclustering reduced the number of heating demand agents from 96 to 20 globally. 96 agents were initially estimated for three geospatial characteristics. However, similarities were observed and a subclustering process was applied to reduce the number of agents. The Elbow Method is used to determine the Optimal Number of Clusters along with the actual final number of clusters per region. The dataset shows the results of measuring agent compactness after applying the subclustering K-means discussed previously. The percentage of well-grouped data [percentage_of_well_grouped_data in dataset] shows the usual decomposition of deviance in deviance between clusters (BSS) and deviance within clusters (TSS). Ideally, the subclustering seeks clusters that have the properties of internal cohesion and external separation. Therefore, the ratio of BSS/TSS approaching 1 represents the compactness of the subclustering of agents^31^. Despite having 96 agents initially, a high percentage of well-grouped data means that the final 20 agents have similar members within each new cluster after the application of the Elbow Method. In summary, if all 96 agents were selected without using the Elbow Method, the BSS/TSS ratio would be 1, thereby achieving 100% compactness. Overall, the separate subclustering conducted for each MUSE-RASA region produced a BSS/TSS ratio greater than 0.975, which means that more than 97% of the initial 96 agents were well grouped into 20 agents. Additionally, the Silhouette coefficient [ave_sil_width in dataset] has been used to evaluate the goodness of the subclustering. Overall, a *Si* greater than zero indicates that the agents are well grouped. The closest *Si* is to 1, the best it is clustered. A *Si* < 0 indicates that agents were placed in the wrong group. In addition, *Si* = 0 indicates that the agents are between two clusters. These two variables are of especial importance for the cross-validation of agent characterisation and further parametrisation.

### Dataset of global geospatial cross-validation errors [12_spatial_cross_validation_errors.csv]

This dataset provides the results of the third validation process in addition to the validation previously discussed. The error between the agent parametrisation values and the aggregated parameter at the regional level is provided in this dataset. Errors have been estimated for GDP, GDPpp, TE, SH, WH, Pop and HDI. Overall, the agent parametrisation approach suggests a global measure of error that is satisfactory, as the error is minimum in most agents and regions.

### Dataset of global region shapes [13_Regions_shapes.shp]

This dataset provides the MUSE-RASA regions used in this research in a geospatial format [. shp]. The 28 regions of the MUSE model are provided, which have been extensively documented in the literature^14^ and^32^.

## Technical Validation: Spatial Cross-Validation

Four validation processes have been conducted in this research to validate the Geospatial Agent-Based Modelling (G-ABM) Framework, including the characterisation of heterogeneity (clustering and subclustering), and the parametrisation of diversity. First, the G-ABM approach was validated by comparing the official values of the two selected countries with those estimated in this study, as published in Sachs, *et al*.^9^. Second, the quality of clustering performed using the spatial K-means algorithm on GDPpc, HD, and HDpc was assessed worldwide. Details of this validation of the spatial characterisation are provided in Moya, *et al*.^28^. Third, the subclustering goodness of the final spatial agents was measured using the Silhouette coefficient (Silhouette width), as can be seen in dataset No. 11. Finally, the error of the diversity parametrisation of each agent attribute was also calculated and provided in dataset No. 12. This was performed by comparing the aggregated agent results with the total regional values.

**Fig. 16:**
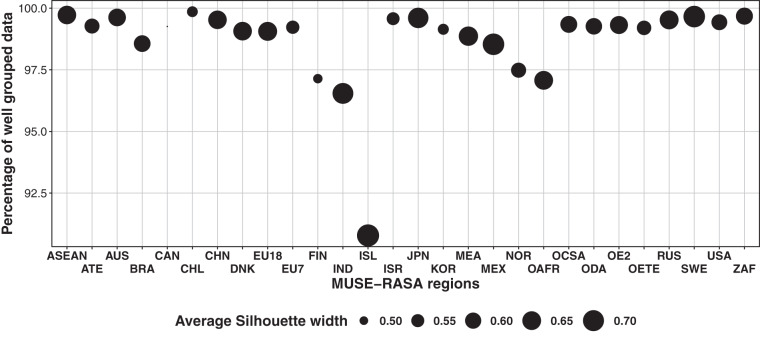
Third validation process of this study. Quality of clustering done using the spatial K-Means algorithm. Quality is assessed by the application of K-Means BSS/TSS ratio. SS = sum of squares. BSS = low similarity between clusters. TSS = total deviance within groups sums of squares. Deviance concept is used instead of Variance concept because BSS/TSS ratio seeks to measure the model fit.

The global number of heating demand agents was reduced from 96 to 20 through the process of subclustering. Figure 16 illustrates the results of measuring the compactness of the agents after applying the subclustering K-Means discussed in the Methodology, in the third validation process conducted in this research. The y-axis in Fig. 16 represents the percentage of well-grouped data, which indicates the division of deviance between clusters (BSS) and within clusters (TSS). Ideally, the subclustering aims to create clusters that exhibit internal cohesion and external separation. Thus, a BSS/TSS ratio approaching 1 explains the compactness of the subclustering of agents^31^. Despite initially having 96 agents, a high percentage of well-grouped data indicates that the final 20 agents share similar members within each new cluster after applying the Elbow Method. In other words, if all 96 agents were chosen without using the Elbow Method, the BSS/TSS ratio would be 1, achieving 100% compactness. In summary, the separate subclustering performed for each MUSE-RASA region resulted in a BSS/TSS ratio greater than 0.975, indicating that over 97% of the initial 96 agents were effectively grouped into 20 agents. This outcome is particularly significant for the subsequent stages of the research, as agent definition involves specific zones of GDPpc, HD, and HDpc (characterization) with a range of parameters (parametrization).

**Fig. 17:**
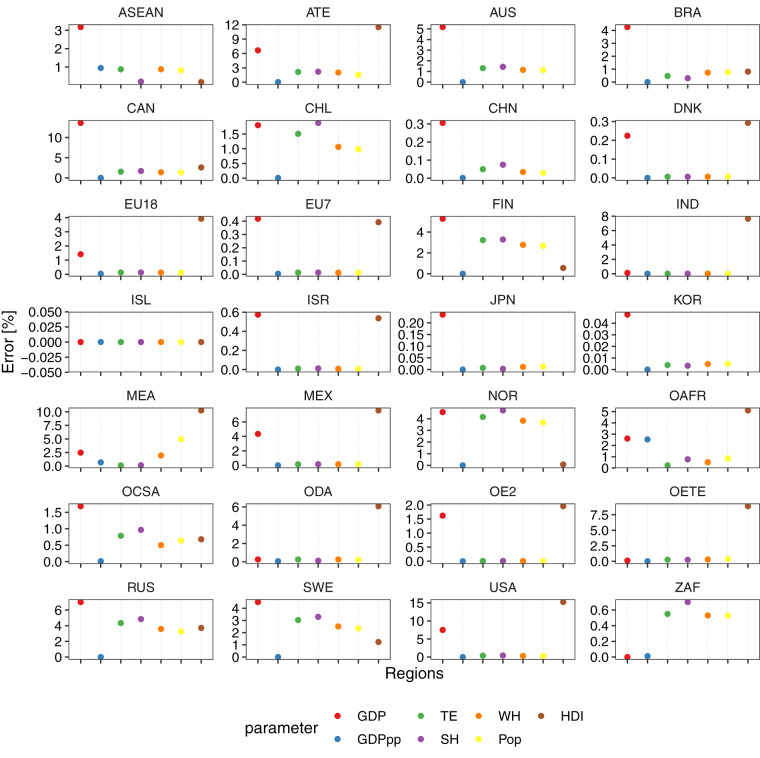
Fourth validation process of this study. Error estimation of the agent parametrisation approach. Estimated values for each agent in each region are aggregated and then compared against aggregated regional values.

Figure 17 depicts the validation process for agent parametrization, which is the fourth validation procedure conducted in this research alongside with the previous spatial-cross validation processes. The figure presents the disparity and comparison between the agent parametrization values from this research and the aggregated parameter at the regional level from data sources. It can be observed that in certain regions (CHN, DNK, EU7, ISL, ISR, JPN, KOR, and ZAF), the error is less than 1%. However, in the case of GDP in CAN, the error can reach 10%, and for HDI in ATE, error can go up to 12%. Overall, the agent parametrization approach demonstrates an acceptable level of global error, as the majority of agents and regions exhibit minimal error.

## Usage Notes

The datasets provided in this study^8^ are of real importance for researchers exploring the combination of GIS with ABM where socioeconomics and energy demands are needed. The datasets are spatially resolved and temporarily explicit, which serve to capture the spatiotemporal dimensions in global model simulations. A range of agents are systematically defined. It is suggested that these datasets be used as inputs in future research on the decarbonisation of the energy system when considering the human dimension.

Stakeholders of the sustainable transition of the climate-energy-economy system can benefit of this research datasets in several manners. Decision-makers, policy-makers, firms, civil society, and researchers can identify four potential applications of these datasets in the context of assessing climate-energy-economy transition paths:

1. **Agent budget limitations:** the datasets presented here^8^ embed intra-regional differences among energy consumers of the residential sector. This has important implications in climate-energy-economy modelling for designing policies, capturing heterogeneities, diversities, evolution, decision-making and external drivers of energy and economic agents in the assessment.
2. **Agents that drive the transition:** this research has identified the main agents that will drive the climate-energy-economy transition globally. These agents are characterised and define with a range of parameters, openly share in this research^8^. Specific agents meet certain and customised characteristics defined by stakeholders to reach defined and designed goals such as changing energy use behaviour or adopting clean-highly efficient technologies.
3. **Carbon tax schemes implementation:** Carbon tax schemes are hard to implement because of the regressive impact on poorer households. This research can contribute towards minimising or eliminating the impact of carbon tax schemes implementation. To accelerate the sustainable transition towards the NZE target by mid-century, this research helps policy-makers and implementers of carbon tax schemes by targeting and focusing on agents that can afford it or developing financial assistance programs for those that are unable to meet such taxes.
4. **Research and development prioritisation based on heat density:** institutions in charge of researching, innovation and development of new solutions for a sustainable transition of the global climate-energy-economy system can also be beneficiaries of the results of this research^8^. Additional applications would apply for consumers living in zones where district heating technologies are technically feasible because of the high energy density observed there.

Limitations and challenges are also identified in this research. The main limitation of this study is the validation of the decision-making process part. Although four systematic spatial cross validation processes have been conducted for the general framework for the spatial agent definition, there is lack of data about agent decision-making processes to validate any agent investment objective use in future assessments. The only way to inform specifically the decision-making process would require targeted surveys in the location, city, country, or region under study. Examples of surveys carried out for China and Ecuador, to collect primary data to characterise the decision-making process, are presented in this research^8^. However, conducting a representative survey for all worldwide regions of this research would be a time- and resources- consuming task. National surveys would enrich the agent disaggregation analysis that this study has proposed. This could apply not only for the residential sector, but also for other sectors such as industry, transport, and agriculture. In this way, the research and datasets here can be applied to other sectors accordingly.

## Data Availability

The algorithms and formulas used in this study have been previously provided. This research used three programmatic free and open-source platforms: (1) R Statistical Software and Programming Language; (2) Quantum GIS (QGIS) software; and (3) Python software. A range of R Packages for geospatial big data analytics used in this research are presented in Bivand^33^. QGIS is used for data exploration purposes because of its features of viewing, editing, and analysing geospatial data^34^. Python is the development programmatic environment for the MUSE model^35^. The MUSE-RASA model has been built from the integration of the R-based geospatial RASA model with a Python-based MUSE model to end with the MUSE-RASA model. The R code used to create the shape files in the RASA model is available upon request with proper justification from the corresponding author. The MUSE model is an open source code available in Giarola, *et al*.^36^. Due to sponsorship agreements, the authors are not allowed to make the RASA code publicly available.
